# Isobavachalcone Induces ROS-Mediated Apoptosis *via* Targeting Thioredoxin Reductase 1 in Human Prostate Cancer PC-3 Cells

**DOI:** 10.1155/2018/1915828

**Published:** 2018-10-16

**Authors:** Kai Li, Qiusheng Zheng, Xiaoyu Chen, Yunchao Wang, Dan Wang, Jianning Wang

**Affiliations:** ^1^Department of Urology, Shandong Provincial Qianfoshan Hospital, Shandong University, Jinan 250014, China; ^2^Binzhou Medical University, Yantai, Shandong 264003, China

## Abstract

Prostate carcinoma causes a great number of deaths every year; therefore, there is an urgent need to find new drug candidates to treat advanced prostate cancer. Isobavachalcone (IBC) is the chalcone composition of *Psoralea corylifolia* Linn used in traditional Chinese medicine. Although IBC demonstrates potent anticancer efficacy in numerous types of human cancer cells, the cellular targets of IBC have not been fully defined. In our study, we found that IBC may induce reactive oxygen species- (ROS-) mediated apoptosis via interaction with a selenocysteine (Sec) containing the antioxidant enzyme thioredoxin reductase 1 (TrxR1), and induce lethal endoplasmic reticulum (ER) stress by inhibiting TrxR1 activity and increasing ROS levels in human prostate cancer PC-3 cells. Furthermore, we also observed that knocking down TrxR1 would sensitized cancer cells to IBC treatment. Our study provides evidence for the anticancer mechanism of IBC with TrxR1 as a potential target.

## 1. Introduction

Prostate cancer leads to the second greatest number of cancer-related deaths following lung cancer. It is also the most frequent cancer diagnosis in men over 60 years of age and has a high incidence rate in younger men as well [1, 2]. However, treatment options for prostate cancer are currently unsatisfactory, where the development of hormonal resistance is essentially inevitable and is the foremost cause of death in prostate cancer patients [3]. Therefore, it is urgent to accelerate the development of potential therapeutic agents to treat hormone refractory prostate cancers. Compounding this, these tumors often become highly resistant to conventionally used cytotoxic agents. Chinese traditional herbal medicine has been used to treat a wide range of diseases, including cancer, for hundreds of years [4]. Many natural compounds kill cancer cells and have no or minimal effects on normal cells. Therefore, there is an urgent search for natural compounds with anticancer activities to treat prostate cancer.

The thioredoxin (Trx) system plays an important role in regulating the redox balance in cells, which is composed of thioredoxin reductase (TrxR), Trx, and nicotinamide adenine dinucleotide phosphate (NADPH) [5–7]. TrxR is the only known enzyme capable of degrading thioredoxin, the inhibition of whose activity may destroy the redox function of some cells and lead to an increase in reactive oxygen species [8]. Oxidative stress is an important regulator of the behavior of cancer cells. While proper physiological reactive oxygen species (ROS) levels are necessary to maintain a number of cellular functions, ROS overproduction can overwhelm available intracellular antioxidants. This can disrupt the prooxidant/antioxidant balance, which can lead to cell damage and death [9, 10]. A series of proapoptotic signaling pathways can also be triggered by overproduction of ROS, including those involved in mitochondrial dysfunction and endoplasmic reticulum (ER) stress, which can eventually result in apoptosis [11]. Malignant cells experience more oxidative stress than normal cells, rendering them vulnerable to oxidative stress [12, 13]. Increasing evidence indicates that increasing oxidative stress may be a means of effectively eliminating cancer cells. Notably, prostate cancer cells have been demonstrated to have increased ROS production and oxidative stress [14]. Therefore, ROS-inducing agents may effectively kill prostate cancer cells.

Abnormal accumulation of unfolded/misfolded proteins or abrupt changes in ER Ca^2+^ homeostasis causes an adaptive response in cells referred to as ER stress. However, excessive ER stress is closely associated with mitochondrial dysfunction and oxidative stress, and can result in apoptotic cell death [15–17]. Therefore, therapeutic modulation of proapoptotic ER stress is a possible means of chemosensitizing hormone refractory prostate cancer cells [18, 19].

Isobavachalcone (IBC) is a chalcone with multiple activities, including antifungal, anti-inflammatory, and antireverse transcriptase [20]. Prior work on a series of tumor and normal cells, including human umbilical vein endothelial and liver cells and the human hepatocyte LO2 line, has demonstrated that IBC has a notable toxicity on tumor cells but not normal cells [21]. This suggests that IBC treatment may be effective and have low toxicity in cancer patients. Reports have been published focusing on the anticancer abilities of IBC in certain types of tumors. However, how IBC affects human prostate cancer and the mechanisms behind these effects have yet to be fully delineated. The present study uses PC-3, a human androgen-independent prostate cancer cell line, to study the inhibition of proliferation by IBC *in vitro*, as well as the mechanisms mediating this inhibition. Overall, this work aims at providing theoretical and experimental evidence for furthering development of a candidate small molecule compound for targeted therapy against androgen-independent prostate cancer.

## 2. Materials and Methods

### 2.1. Chemicals and Reagents

IBC (purity ≥ 98%) was obtained from Lichen Biotechnology Co. Ltd. (Shanghai, China). Dimethylsulfoxide (DMSO), buthionine sulfoximine (BSO), N-acetylcysteine (NAC), annexin V/PI apoptosis kit, Hoechst 33258, and 2′,7′-dichlorofluorescein diacetate (DCFH-DA) were purchased from Sigma-Aldrich (St. Louis, MO, USA). Streptomycin and penicillin were purchased from Sunrise Pharmaceutical Co. Ltd. (Shandong, China). IBC was initially dissolved in DMSO to obtain a stock solution and then diluted in RPMI 1640 to obtain a working solution with a final DMSO concentration of less than 0.1%, which does not adversely affect cell viability. Any other reagents were purchased from Sigma Chemical Company (St. Louis, Missouri, USA).

### 2.2. Cells and Cell Culture

The human prostate cancer PC-3 cell line was purchased from the ATCC. Cells were cultured in RPMI 1640 containing 10% FBS, 100 U/mL penicillin, and 100 *μ*g/mL streptomycin in a humidified incubator with 5% CO_2_ at 37°C.

### 2.3. Treatments with Drugs

Immediately before each experiment, the IBC stock solution was diluted in culture media. IBC working solutions had a DMSO concentration of less than 0.1%, and RPMI 1640 containing an equal concentration of DMSO as the IBC working solution was used as the control. In the first part of this study, PC-3 cells were treated with 15, 30, or 45 *μ*M IBC. In the second part of this study, PC-3 cells were treated with 45 *μ*M IBC in the absence or presence of 5 mM NAC and 1 mM BSO. Following treatment, cells were harvested for further analysis.

### 2.4. Cell Viability

Cell viability was measured using the MTT assay. First, 96-well plates were seeded with PC-3 cells at a density of 0.7 × 10^4^ cells per well and incubated for 24 h at 37°C. These cells were then treated with IBC at different concentrations for 24, 48, or 72 h. Then MTT (5 mg/mL) solution was added to each well and incubated for 4 h. DMSO was added to each well (150 *μ*L/well) and the absorbance at 490 nm was measured using a microplate reader (Bio-Rad Laboratories, Hercules, CA, USA).

### 2.5. Morphology

To examine apoptosis of PC-3 cells, 2 × 10^5^ cells per slide were seeded on 6-well chamber slides and treated with 15, 30, or 45 *μ*M IBC for 24 h. An inverted phase contrast microscope was used to examine cell morphology and images were obtained using a 600D camera (Canon Inc., Japan). Cells were also fixed in 40 g/L formaldehyde in PBS for 20 min and then stained with 10 mg/L Hoechst 33258 for 30 min in the dark. The stained cells were visualized using fluorescence microscopy [22].

### 2.6. Measurement of Apoptotic Population

Apoptosis was measured by staining cells with annexin V-FITC and PI. [23] Briefly, cells were incubated with IBC at a series of concentrations for different periods of time. Then, the cells were washed and stained with 5 *μ*L each of annexin V-FITC and PI for 10 min, followed by analysis with flow cytometry (BD, NJ, USA).

### 2.7. Measurement of Intracellular ROS Levels

Intracellular ROS concentrations were quantified by flow cytometry as previously described [24]. Briefly, 3 × 10^5^ cells were seeded in 60 mm dishes, incubated overnight, and then treated with reagents for different periods of time. Then, 10 *μ*M DCFH-DA (Beyotime Institute of Biotechnolgy, Nantong, China) was added to the cells and incubated at 37°C for 30 min. The cells were then collected, washed, and the fluorescence evaluated using flow cytometry (BD, NJ, USA).

### 2.8. Quantitative Real-Time Polymerase Chain Reaction

Total RNA was purified from PC-3 cells using TRIzol Reagent (Invitrogen) and used as template for reverse transcription using the PrimeScript RT-PCR Kit (TaKaRa, China). The resulting cDNA was used in semiquantitative real-time PCR. *β*-Action was used as an endogenous reference. PCR primer sequences were as follows: human GRP78, forward 5′-ACTGTTACAATCAAGGTCTATGAAGG-3′ and reverse 5′-CAAAGGTGACTTCAATCTGTGG-3′; human XBP-1, forward 5′-GCGCCTCACGCACCTG-3′ and reverse 5′-GCTGCTACTCTGTTTTTCAGTTTCC-3′; human ATF4, forward 5′-TGGCTGGCTGTGGATGG-3′ and reverse 5′-TCCCGGAGAAGGCATCCT-3′; human CHOP, forward 5′-CAGAACCAGCAGAGGTCACA-3′ and reverse 5′-GCTGTGCCACTTTCCTTTC-3′; and human *β*-action, forward 5′-TCCTTCCTGGGCATGGAGTC-3′ and reverse 5′-GTAACGCAACTAAGTCATAGTC-3′.

### 2.9. Western Blot

Cells (1.0 × 10^5^ cells/mL) were treated with IBC (15, 30, or 45 *μ*M) for 24 h. After homogenization in protein lysate buffer, cells were centrifuged at 12,000 rpm at 4°C for 10 min. Protein concentrations of samples were measured and loading buffer was added to the protein samples, which were then electrophoresed and transferred to polyvinylidene difluoride transfer membranes. After blocking with fresh 5% nonfat milk in TBST for 2 h at room temperature, the blots were incubated with primary antibody diluted in TBST at 4°C overnight. The blots were washed three times with TBST and then incubated with horseradish peroxidase-conjugated secondary antibody for 1 h. Immunoreactive bands were visualized using an ECL kit (Bio-Rad Laboratories, Hercules, CA, USA) and the density of these bands was determined using Image J software (National Institute of Health, MD).

### 2.10. *In Vitro* TrxR Activity Assays

A microplate reader (SpectraMax M5, Molecular Devices, USA) was used to determine TrxR activity by DTNB assay at room temperature. The incubation of NADPH-reduced TrxR (150 nM) was achieved with IBC of different concentrations for two hours at room temperature (with the eventual volume of the reaction mixture as 50 *μ*L) on a 96-well plate. The master mixture of TE buffer (50 mM Tris-HCl, pH 7.5, 1 mM EDTA, 50 *μ*L) containing DTNB and NADPH (final concentrations: 2 mM and 200 *μ*M, respectively) was added. The record of linear addition in absorbance at 412 nm during the first four minutes was performed. The equal amount of DMSO (1%, *v*/*v*) was added in the control experiments with the activity denoted as percentage of the subjects in the control experiments.

### 2.11. Determination of TrxR Activity in Cells

After the treatment of cells with IBC with different concentrations for two hours, the harvesting and extraction of cells were achieved with RIPA buffer. The Bradford protein assay kit (Bio-Rad Laboratories, Hercules, CA, USA) was applied to determine the content of total protein. Based on an insulin decrease assay at the end points, the measurement of TrxR activity in cell lysates was achieved. Soon, incubation of cell extract was achieved and the eventual reaction volume was 50 *μ*L, containing 100 mM Tris-HCl (pH 7.6), 0.3 mM insulin, 660 *μ*M NADPH, 3 mM EDTA, and 15 *μ*M *E. coli* Trx (Sigma-Aldrich, St. Louis, MO), for a duration of 30 min at 37°C. A total of 40 *μ*g of protein were contained in the extract. Through the addition of 200 *μ*L of 1 mM DTNB in 6 M guanidine hydrochloride (pH 8.0), the termination of the reaction was achieved. A blank sample, containing everything except Trx, was treated in the same manner.

The measurement of absorbance at 412 nm was carried out, followed by the subtraction of the blank value from the absorbance value of the sample accordingly. The activity was denoted as the percentage of the subject in the control group.

### 2.12. Transient Transfection of Small Interfering RNA (siRNA)

Small interfering RNA molecules, specifically targeting the TrxR1 mRNA, were obtained from Sigma-Aldrich (St. Louis, MO). For phenotypic confirmation, the following sequence was used: sense 5′-(CUUUGCAGCUGCGCUCAAA)dTdT-3′, antisense 5′-(UUUGAGCGCAGCUGCAAAG)dTdT-3′. The siRNA duplexes targeting TrxR1 were transduced into PC-3 cells. Forty-eight hours after transduction, cells were washed with complete media and seeded on 6-well plates. After treatment with IBC for 12 h or 24 h, cells were harvested for immunoblot (12 h) or apoptosis (24 h) analysis.

### 2.13. Statistical Analysis

Results from at least three independent experiments are presented as mean ± S.D. Statistical significance was determined using analysis of variance followed by Student's *t*-test. A *p* value of <0.05 was considered statistically significant. All statistical analyses were performed using GraphPad Prism Pro 6.0 (GraphPad, San Diego, CA).

## 3. Results

### 3.1. IBC Inhibits Proliferation of PC-3 Cells

**Figure 1:**
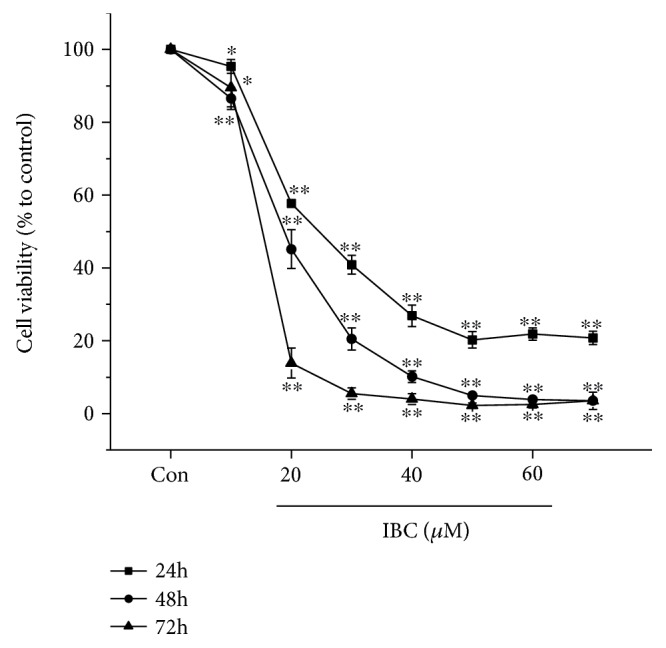
Effect of IBC on the proliferation of PC-3 cells. After treatment with IBC at a series of concentrations for 24, 48, or 72 h, the viability of PC-3 cells was assessed with an MTT assay. All the data are expressed as percent change compared to the control group, which was arbitrarily designated as having 100% viability. All data are mean ± S.D. ^∗^*p* < 0.05 and ^∗∗^*p* < 0.01 compared to the control group.

PC-3 cells were cultured with 0–70 *μ*M IBC for 24, 48, or 72 h, followed by analysis with methyl thiazolyl tetrazolium (MTT) assay. The results showed that IBC can time- and concentration-dependently inhibit the proliferation of PC-3 cells (Figure 1). The 50% inhibitory concentrations (IC50s) of IBC in PC-3 cells for treatment for 24, 48, and 72 h were approximately 26.19, 19.25, and 14.80 *μ*M, respectively.

### 3.2. IBC Induces Apoptosis of PC-3 Cells

**Figure 2:**
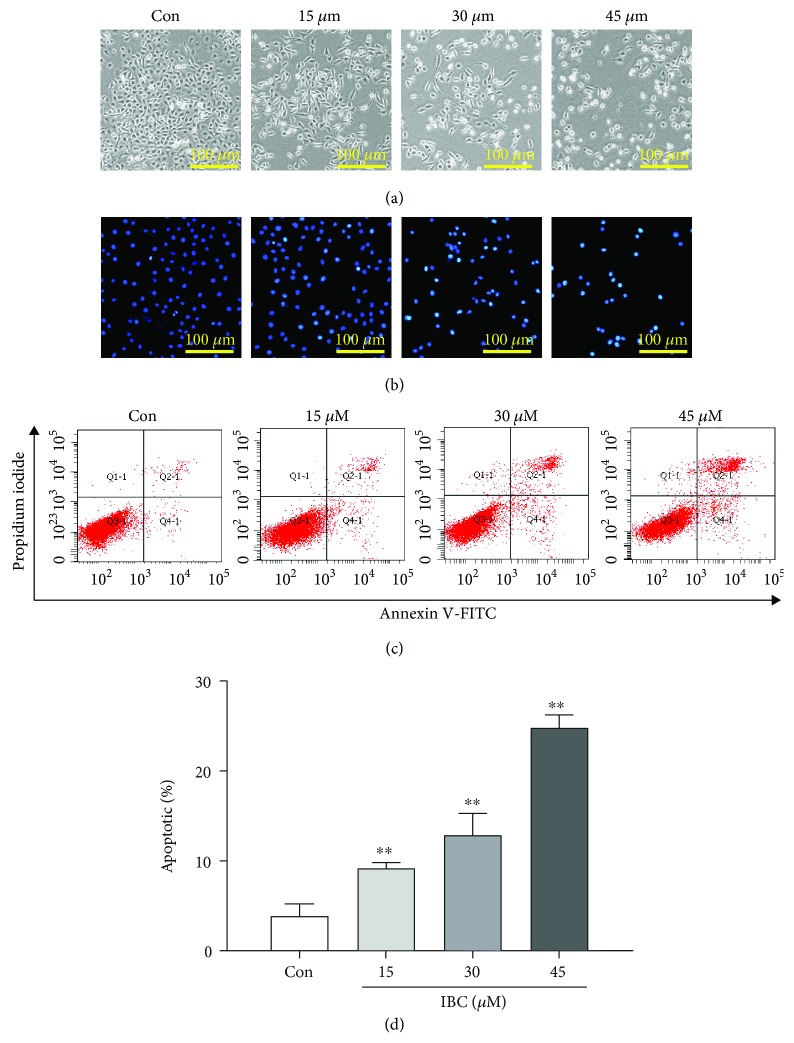
Effect of IBC treatment on PC-3 cell morphology and apoptosis. Cells were exposed to 15, 30, or 45 *μ*M IBC for 24 h. (a) After treating cells for 24 h, the morphology of PC-3 cells was visualized and imaged by inverted phase contrast microscopy. (b) The morphological changes in PC-3 cells were examined following staining with Hoechst dye 33258. (c) Rate of apoptotic cells of IBC-treated PC-3 cells as detected by flow cytometry. (d) Quantitative analysis of the rate of apoptotic cells after IBC treatment. All data are presented as mean ± S.D. ^∗^*p* < 0.05 and ^∗∗^*p* < 0.01 compared with the control group.

Cells undergoing apoptosis characteristically present with cell shrinkage, membrane blebbing, nuclear condensation, and cleavage of chromatin. Therefore, apoptosis can be identified by light and electron microscopy based on the distinct apoptotic cell morphology [25]. Microscopic visualization of cell morphology revealed that IBC treatment caused notable cell shrinkage based on the scale bar and gaps between cells and reduced attachment of cells based on the cell number compared to the control group (Figure 2(a)).

Morphology was examined to quantify apoptosis using Hoechst dye staining. As shown in Figure 2(b), IBC-treated cells presented with apoptosis-associated characteristics, including small vesicles, cell shrinkage, chromatin compaction, cytoplasm condensation, and nuclear fragmentation, where the nuclei were smaller and more fluorescent than those in the control cells. Meanwhile, phosphatidylserine externalization, which is a hallmark of early apoptosis, was assessed by double staining cells with PI and annexin V-FITC. Using flow cytometry, it was found that IBC significantly increased the percent of apoptotic cells from 3.87% in the control group to 24.8% in the 45 *μ*M IBC treatment group (Figures 2(c) and 2(d)).

### 3.3. Effects of IBC on ER Stress Signaling and Apoptosis-Related Factors in PC-3 Cells

**Figure 3:**
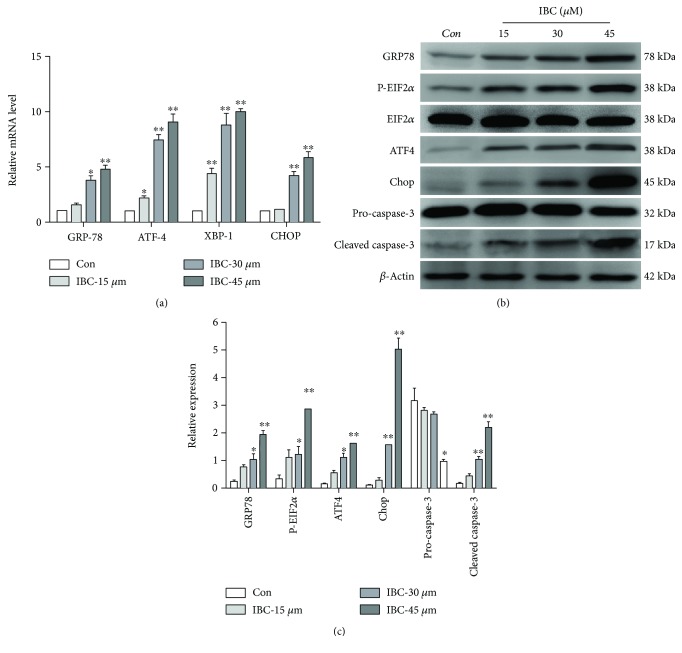
IBC activates ER stress, which contributes to the IBC-induced lethality of PC-3 cells. Cells were exposed to 15, 30, or 45 *μ*M IBC for 24 h. (a) Quantitation of levels of GRP78, XBP-1, CHOP, and ATF4 mRNA by RT-qPCR. (b) Protein levels of GRP78, p-eIF2*α*, ATF4, Chop, eIF2*α*, pro-caspase-3, and cleaved caspase-3 were determined by Western blot. (c) Quantitation of GRP78, p-eIF2*α*, eIF2*α*, ATF4, Chop, pro-caspase-3, and cleaved caspase-3 protein levels. All data are presented as mean ± S.D. ^∗^*p* < 0.05 and ^∗∗^*p* < 0.01 compared to the control group.

The next aim is to explore the mechanisms underlying the anticancer effects of IBC. Therefore, the effect of IBC treatment on the induction of ER stress was examined. RT-PCR revealed that treatment with IBC led to the upregulation of GRP78, ATF4, XBP-1, and Chop mRNA levels in PC-3 cells in a dose-dependent manner (Figure 3(a)). Next, the expression of a series of ER stress-related proteins including ATF4, GRP78, Chop, and p-eIF2*α* was measured in PC-3 cells with or without IBC treatment. Results showed that IBC treatment induced a significant increase in protein expression levels. Specifically, p-eIF2*α* levels peaked when treated with 15 *μ*M IBC (Figures 3(b) and 3(c)) and 45 *μ*M IBC significantly activated ER stress. In addition, expression levels of downstream effectors in apoptosis were measured. As shown in Figure 3(b), treating cells with IBC for 24 h activated caspase-3 cleavage in a concentration-dependent manner, suggesting that apoptosis of PC-3 cells induced by IBC may be associated with the activation of the caspase pathway. This evidence demonstrates that IBC-induced apoptosis of PC-3 cells is mediated at least partially by the ER stress pathway.

### 3.4. IBC Directly Binds and Inactivates TrxR1 in PC-3 Cells

At present, TrxR has been considered as an important target molecule for the development of anticancer drugs [26]. Among the known TrxR inhibitors, the most common is the compound with a Michael addition receptor, which can be covalently bonded with the TrxR carbon-end Sec to reduce its activity [8, 27–29]. Isobavachalcone contains a *α*,*β*-unsaturated ketone structural unit, which is likely to inhibit TrxR activity by having a covalent additive reaction with the TrxR. When the TrxR is suppressed to lose its antioxidant function, the level of ROS in the cell increased and the intracellular redox balance was broken, resulting in oxidative stress.

**Figure 4:**
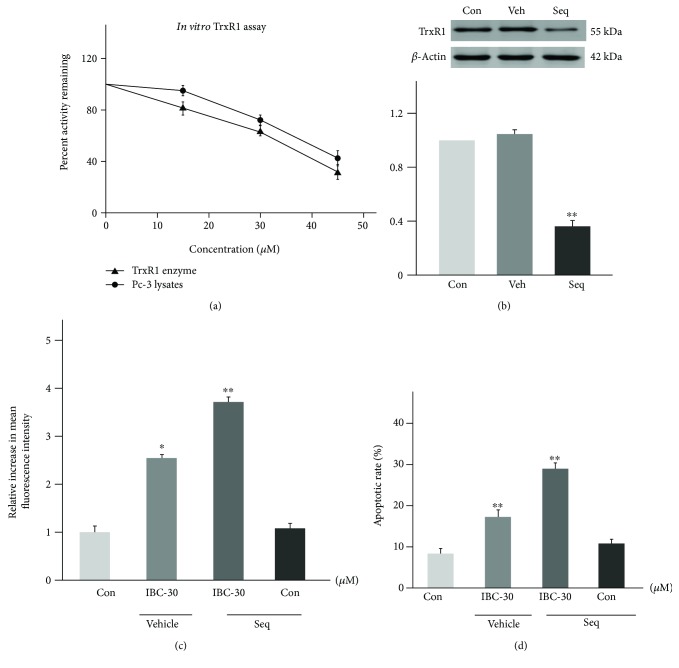
IBC directly binds and inactivates TrxR1 in PC-3 cells. (a) The activity of the TrxR1 enzyme. (b) The TrxR1 expression was determined by Western blotting after knockdown with siRNAs for 12 h. (c) Knockdown of TrxR1 in PC-3 cells significantly increased the ROS levels (d) and apoptotic cells. All data are presented as mean ± S.D. ^∗^*p* < 0.05 and ^∗∗^*p* < 0.01 compared to the control group.

As shown in Figure 4(a), IBC can inhibit the enzyme catalytic activity of the TrxR1 protein in a concentration-dependent manner, whether to the cell lysate or directly to the recombinant TrxR1 protein. The above results show that IBC can inhibit the activity of the TrxR1 protein, but whether TrxR1 mediates the activity of IBC against prostate cancer PC-3 cells needs further verification. Therefore, the following test examined the effect of TrxR1 silence on IBC-induced apoptosis of prostate cancer cells. As shown in Figure 4(b), the constructed TrxR1-specific siRNA can significantly silence the expression of the TrxR1 protein. Further research shows that TrxR1 silence can increase IBC-induced ROS level (Figure 4(c)) and apoptosis of prostate cancer cells (Figure 4(d)), indicating that TrxR1 participates in IBC's anti-prostate cancer effect.

### 3.5. Apoptosis of PC-3 Is Mediated by IBC-Induced ROS Overproduction

**Figure 5:**
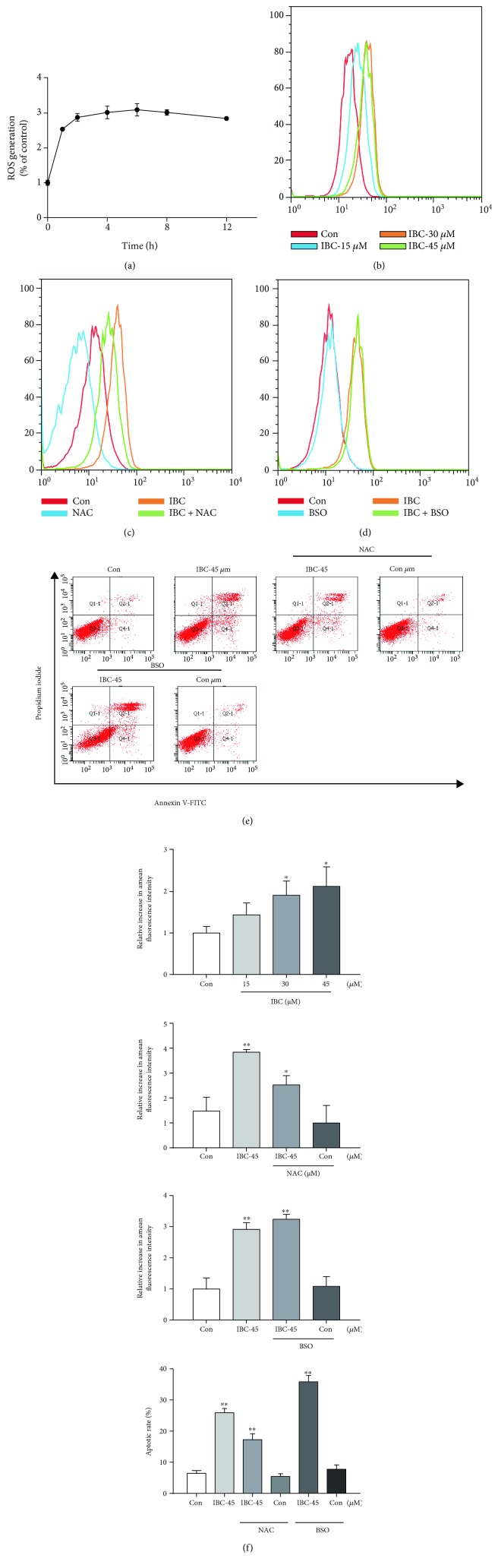
IBC-induced PC-3 cell apoptosis is dependent on intracellular ROS generation. (a) Time course of IBC-induced ROS production. As indicated, cells were treated for different lengths of time with 45 *μ*M IBC and then stained with 10 *μ*M DCFH-DA. The fluorescence intensity was measured by flow cytometry. (b) Intracellular IBC-induced ROS generation was measured by flow cytometry. (c-d) Representative images of ROS production in cells treated with IBC in the presence or absence of NAC or BOS. PC-3 cells were preincubated with NAC or BSO for 2 h before incubating with 45 *μ*M IBC for 6 h. Intracellular ROS production was measured by flow cytometry. (e) Representative images of apoptotic cells identified by staining with annexin V-FITC/PI. Cells were treated with 45 *μ*M IBC in the presence or absence of NAC or BSO for 24 h. Percentage of apoptotic cells was determined by annexin V/PI staining and flow cytometry. (f) Quantitative analysis of the rates of apoptotic PC-3 cells after IBC treatment. Data from three independent experiments are presented as mean ± SD. ^∗^*p* < 0.05 and ^∗∗^*p* < 0.01.

Accumulating evidence suggests that certain therapeutic agents trigger ROS production and this ROS production contributes to their mechanisms of cytotoxicity [30–32]. However, there have been few reports on IBC-induced ROS generation and oxidative stress. Therefore, generation of intracellular ROS with or without IBC treatment was measured by flow cytometry. Figures 5(a) and 5(b) show that IBC (45 *μ*M) time- and concentration-dependently induced a significant increase in intracellular ROS in PC-3 cells. To elucidate the role of ROS in the anticancer effects of IBC, cells were treated with 5 mM of antioxidant NAC and 1 mM of the prooxidant buthionine sulfoximine (BSO) for 2 h prior to treatment with IBC, which did not significantly affect basal levels of cellular proliferation. ROS levels decreased in cells cotreated with NAC and IBC and increased in cells cotreated with BSO and IBC (Figures 5(c) and 5(d)). Similar results were noted in assays assessing apoptosis using flow cytometry (Figure 5(e)). Overall, these results confirm that IBC-induced apoptosis of PC-3 is mediated by ROS production.

### 3.6. IBC-Induced ER Stress in PC-3 Cells Is Mediated by ROS Generation

**Figure 6:**
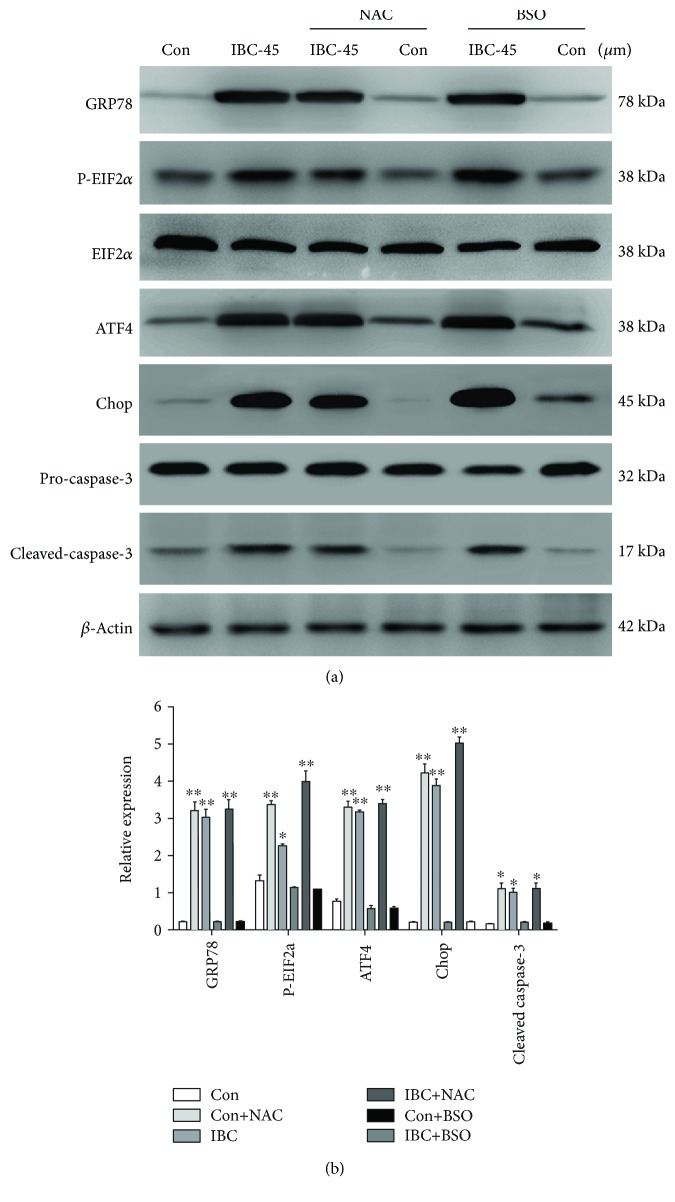
IBC-induced ER stress was mediated by ROS generation. PC-3 cells were treated with 45 *μ*M IBC with or without 5 mM NAC or 1 mM BSO for 24 h, and then ER stress marker expression levels were measured by Western blot. (a) Protein levels of GRP78, ATF4, Chop, and p-eIF2*α* were examined by Western blot. (b) Quantitative analysis of GRP78, ATF4, Chop, and p-eIF2*α* protein levels. All data are presented as mean ± S.D. ^∗^*p* < 0.05 and ^∗∗^*p* < 0.01 compared to the control group.

In this study, we discovered that ROS generation activated the proapoptotic signaling pathway, including the cell apoptosis pathway induced by ER stress [33, 34]. IBC-induced ROS generation occurs at a much earlier timepoint (1 h) than ER stress activation. Therefore, ROS production may occur upstream in IBC-induced cell death in PC-3 cells. Next, we characterized whether ROS production is necessary for IBC-induced ER stress in PC-3 cells. A combined treatment with NAC and IBC notably inhibited IBC-induced ER stress marker overexpression (Figures 6(a) and 6(b)). Conversely, cotreatment with BSO and IBC promoted IBC-induced ER stress marker expression. These results indicate that ER stress induced by treatment with IBC is mediated by increased oxidative stress.

## 4. Discussion

Prostate cancer is a leading cancer in men. Over the past two decades, the 5-year rate of survival has increased due to advancements in diagnostics for and treatment of prostate cancer [35]. However, even aggressive interventions, such as radiotherapy, chemotherapy, and surgery, do not guarantee that prostate cancer patients will be cured, especially those with castration-resistant prostate cancer [36]. Therefore, there is an urgent search for natural compounds with anticancer activities to treat prostate cancer. In this study, we demonstrated that IBC treatment caused apoptotic features, including increased ROS production in apoptotic cells, phosphatidylserine externalization in cell membranes, and caspase-3 activity, as well as a reduction in cell viability, in a concentration- and time-dependent manner. We next further characterize this inhibitory activity of IBC and explore the underlying mechanism in PC-3 cells.

Trx serves as an important target in cancer drug therapy. Trx and TrxR have been proved to be overexpressed in various cancer cells, and the rapid growth of their tumor cells are related to resistance to drugs [37]. Therefore, people are more and more interested in discovering new TrxR inhibitors as potential antitumor drugs [26, 34]. For the first time, we found that TrxR serves as the target of IBC in PC-3 cells, in which IBC can specifically target TrxR and induce apoptosis in PC-3 cells. The inhibiting TrxR stops the reduction of Trx and reduces the activity of various antioxidant enzyme systems that require reduction-state Trx as their electron donor. As a result, it causes the accumulation of ROS and changes the redox state within the cell [5]. The reduction-state Trx directly reacts with various enzymes related to apoptosis, such as ASK1 and pro-caspase-3 [38], and stops apoptosis. Therefore, the inhibition of TrxR strengthens apoptosis. The inhibition of TrxR will result in the generation of SecTRAPs (the Sec of its active site is replaced by an electrophile) [39]. These SecTRAPs have lost the ability to reduce Trx, and it will ultimately result in the rise of oxidative stress within the cell.

Intracellular ROS can cause oxidative stress, can disrupt the balance between prooxidants and antioxidants, and is a common response to cellular damage and death [40]. Results from this present study suggest that ROS has a critical role in IBC-induced ER stress in PC-3 cells. Firstly, ROS generation is concentration-dependently triggered by IBC. Second, ROS scavengers suppress ROS generation, while prooxidants promote ROS production. Third, cotreatment of PC-3 cells with IBC and the ROS scavenger NAC caused a notable inhibition of IBC-induced ER stress marker overexpression, while cotreatment with IBC and the prooxidant BOS had the opposite effect. Overall, this suggests that ROS acts as a signal upstream in the initiation of IBC-induced ER stress. IBC-induced cytotoxicity and stimulation of ER stress in PC-3 cells were decreased, but not completely inhibited, by NAC, which suggests that additional signaling molecules/pathways might contribute to IBC-dependent prostate cancer cell death. A previous work has demonstrated that mitochondrial signaling pathways are involved in the regulation of IBC-induced prostate cancer cell death [21].

The ER is a well-known regulator of cellular responses to stress. Specifically, a disturbance of ER homeostasis triggers the unfolded protein response. Misfolded proteins can accumulate in the ER, which causes ER stress and ultimately results in cell apoptosis [41]. Three major ER stress sensors help protect against the negative effects of ER stress by triggering the unfolded protein response, including ATF6, IRE-1/XBP-1, and PERK/eIF2*α*, which leads to a decrease in translation, degradation of misfolded proteins, and increased levels of chaperones in an effort to increase protein folding and degradation in the ER [42, 43]. CHOP is a downstream player in ER stress and is a marker for cells committed to ER stress-induced apoptosis. CHOP is present at low levels under physiological conditions, but its expression is dramatically increased in response to ER stress at the transcriptional level via the XBP-1, ATF4, and ATF6 pathways [44, 45]. ER stress-induced apoptosis is an attractive signaling target in cancer cells when designing and developing cancer therapeutics. For example, ER stress-mediated apoptosis has been shown to be induced in cancer cells by certain anticancer agents, including EF24, Icariin, and arsenic trioxide [46, 47]. However, the role of ER stress-related apoptotic signals in IBC-induced prostate cancer cell death has yet to be delineated. In this present study, analysis by RT-PCR and Western blot revealed that exposing PC-3 cells to 45 *μ*M IBC significantly increased mRNA and protein expression levels, respectively, of GRP78, phosphorylated eIF2*α*, ATF-4, XBP-1, and CHOP. Collectively, the results of this study underscore the important role that ER stress plays in IBC-induced apoptosis of prostate cancer cells.

Apoptosis has been reported to be essential to the maintenance of cell and tissue homeostasis [48]. In this study, IBC was found to trigger apoptotic events, including phosphatidylserine externalization, chromatin condensation, and DNA fragmentation in PC-3 cells. Activation of caspase-3, the primary executioner caspase in the majority of systems, irreversibly commits a cell to apoptosis. Therefore, caspase-3 activation serves as a reliable marker of apoptotic cells [49]. IBC was found to significantly reduce pro-caspase-3 levels with a concurrent increase in cleaved caspase-3 levels in PC-3 cells. IBC showed high toxicity to PC-3 cells mainly because IBC induced apoptosis, thus enhancing the clinical application of IBC in the treatment of cancer.

## 5. Conclusion

In summary, we have discovered that IBC induces cell apoptosis through ROS-mediated ER stress by interaction with TrxR1. This provides a deep insight into the action mechanism of IBC, and also suggests that TrxR1 is a potential target for the development of anticancer agents.

## Data Availability

The data used to support the findings of this study are available from the corresponding author upon request.
